# Resolution of volatile fuel compound profiles from *Ascocoryne sarcoides*: a comparison by proton transfer reaction-mass spectrometry and solid phase microextraction gas chromatography-mass spectrometry

**DOI:** 10.1186/2191-0855-2-23

**Published:** 2012-04-05

**Authors:** Natasha D Mallette, W Berk Knighton, Gary A Strobel, Ross P Carlson, Brent M Peyton

**Affiliations:** 1 Department of Chemical and Biological Engineering, Montana State University, Bozeman MT 59717, USA; 2 Center for Biofilm Engineering, Montana State University, Bozeman MT 59717, USA; 3 Department of Chemistry and Biochemistry, Montana State University, Bozeman MT 59717, USA; 4 Department of Plant Sciences, Montana State University, Bozeman Montana 59717, USA

**Keywords:** Biofuel, Solid phase microextraction, Proton transfer reaction-mass spectrometry, Volatile organic compounds, Fungal hydrocarbons, Gas chromatography-mass spectrometry

## Abstract

Volatile hydrocarbon production by *Ascocoryne sacroides *was studied over its growth cycle. Gas-phase compounds were measured continuously with a proton transfer reaction-mass spectrometry (PTR-MS) and at distinct time points with gas chromatography-mass spectrometry (GC-MS) using head space solid phase microextraction (SPME). The PTR-MS ion signal permitted temporal resolution of the volatile production while the SPME results revealed distinct compound identities. The quantitative PTR-MS results showed the volatile production was dominated by ethanol and acetaldehyde, while the concentration of the remainder of volatiles consistently reached 2,000 ppbv. The measurement of alcohols from the fungal culture by the two techniques correlated well. Notable compounds of fuel interest included nonanal, 1-octen-3-ol, 1-butanol, 3-methyl- and benzaldehyde. Abiotic comparison of the two techniques demonstrated SPME fiber bias toward higher molecular weight compounds, making quantitative efforts with SPME impractical. Together, PTR-MS and SPME GC-MS were shown as valuable tools for characterizing volatile fuel compound production from microbiological sources.

## Introduction

*Ascocoryne sarcoides *is an endophytic fungus recently isolated from Northern Patagonia and has the potential to produce a petroleum-like fuel (Mycodiesel^®^) directly from a cellulose fermentation process [Strobel, Knighton, Kluck, Ren, Livinghouse, Griffin, Spakowicz and Sears 2008]. Fungi produce medium to long chain hydrocarbons, and the prevalence of these compounds is in the C_19_-C_30 _chain length [Ladygina, Dedyukhina and Vainshtein 2006]. In contrast, *Ascocoryne sarcoides *(NRRL 50072) has been reported to produce a series of straight chained and branched medium chain-length hydrocarbons of C_5_-C_10 _chain length, in the range of gasoline fuel, including heptane, 2-pentene, octane, 1-methyl-cyclohexene, 3,5-octadiene, and cyclodecene [Solomons and Fryhle 2002,Griffin, Spakowicz, Gianoulis and Strobel 2010]. This interesting metabolism requires further work to characterize growth patterns and develop the organism for potential biofuel applications.

Recent studies have identified a variety of compounds produced by *A. sarcoides *on different media using headspace GC-MS on discrete solid phase microextraction (SPME) fiber samples [Griffin, Spakowicz, Gianoulis and Strobel 2010,Strobel et al. 2008]. The SPME technique was introduced in the 1990's [Arthur and Pawliszyn 1990], and SPME fibers have been used to identify and quantify complex volatile mixtures including hydrocarbons from water, soil, food and wine [Langenfeld et al. 1996,Mallouchos et al. 2002,Parkerton et al. 2000,Robinson et al. 2011]. Its advantages include minimizing systematic errors with extraction of volatiles, eliminating the need for solvents [Wercinski 1999], and being simple, fast and relatively sensitive [Pawliszyn 1999]. However, several studies have revealed instabilities with quantification by this method [Brás et al. 2011,Weldegergis et al. 2011,Zeng and Noblet 2002], so further evaluation of the quantitative use of the method for volatile mixtures from *A. sarcoides *was tested.

Another analytical method with potential for analyzing biofuels, proton transfer reaction-mass spectrometry (PTR-MS) has been used to monitor volatile organic compounds (VOCs) emitted from plants, bacteria and subterranean fungi [Aprea et al. 2007,^2009^,Bäck et al. 2010,Bunge et al. 2008,Kai et al. 2010,Maleknia et al. 2007,Ramirez et al. 2009,Steeghs 2004]. These studies often employed solid substrata, rarely included submerged liquid fungal cultivation [Bäck et al. 2010], and collected data in discrete time points. To our knowledge, however, no studies have used PTR-MS to investigate the continuous evolution of volatile organic fuel compounds from a submerged liquid fungal culture over its entire growth cycle.

An advantage of PTR-MS is that it can be run in real time to continuously collect data on the concentrations of specific compounds [Ezra et al. 2004,Lindinger et al. 1998,Tomsheck et al. 2010]. PTR-MS is primarily a quantitative tool and generally requires a detailed knowledge of the components present to be successfully employed. Ion mass (as measured by a quadrupole mass spectrometer) is not a unique indicator of chemical composition. For instance, an ion at m/z 107 can represent either C_8_H_10_H^+ ^(ethylbenzene or xylene isomer) or C_7_H_6_OH^+ ^(benzaldehyde). Beyond the identification of a few small molecules, such as acetaldehyde and methanol, interpretation of the PTR-MS measurements of complex VOC mixtures benefits from the support of another separation technique to provide identification of components.

SPME GC-MS has been combined with PTR-MS to better elucidate VOCs from plants [Aprea et al. 2009,Bouvier-Brown et al. 2007,Ruth et al. 2005] and human lungs [Bajtarevic et al. 2009,King et al. 2010]. These studies showed the combined techniques allowed for both qualitative and quantitative determinations of VOCs. The use of SPME in conjunction with PTR-MS added qualitative value to the interpretation of the PTR-MS data and strengthened the information obtained from experimentation, particularly as it applied to biological metabolic processes. The knowledge of compounds present obtained by SPME allowed elucidation of the most probable sources assigned to ions measured by the PTR-MS.

Here, PTR-MS was combined with headspace SPME GC-MS to explore the applicability of both methods for time-course resolution and quantification of volatiles emitted over the culture cycle of *A. sarcoides*. We present the results of the continuous PTR-MS method as compared with those of discrete headspace SPME GC-MS for monitoring the activity of a novel fungal culture and show both the benefits and shortcomings of each technique.

## Materials and methods

### Culture medium and conditions

*Ascocoryne sarcoides *(NRRL 50072) was provided by Dr. Gary Strobel and grown in a Biostat B Twin Plus 5-liter jacketed reactor (Sartorius Stedim Biotech) on a minimal medium consisting of (per liter): glucose (20 g), ammonium chloride (5 g), NaH_2_PO_4_^.^2H_2_O (2.75 g), MgSO_4_^.^7H_2_O (0.86 g), Ca(NO_3_)_2_^.^4H_2_O (.28 g), yeast extract (0.05 g), and trace salts: KCl (60 mg), KNO_3 _(80 mg), FeCl_3 _(2 mg), MnCl_2 _(5 mg), ZnSO_4 _(2.5 mg), H_3_BO_3 _(1.4 mg), and KI (0.7 mg). The minimal medium was used, in contrast to a more nutrient rich one, to minimize the amount of volatile compounds originating from the medium. An un-inoculated control reactor was run to account for background VOCs resulting from the medium constituents or air supply. Both vessels were sparged with air from compressed air cylinders at 1.5 L/min. This sparge air was humidified prior to introduction into the Biostat to minimize evaporative losses. The pH was controlled at 4.5 with 2 M HCl, 2 M NaOH and was continuously monitored by an internal Hamiltion Easyferm Plus probe. In addition, the stir rate (250 rpm) and temperature (23°C) were held constant by the Biostat control system. The reactor gaseous effluent was sent to the PTR-MS and an off-gas analyzer for O_2 _and CO_2 _measurements.

Accumulation of *A. sarcoides *was analyzed through daily samples of cell dry weight, where samples were filtered onto glass fiber filters (Whatman GF/F), rinsed, and dried overnight in an oven at 80°C. Once removed from the oven, filters were allowed to equilibrate at room temperature in a desiccator and then weighed. Daily glucose concentrations were measured by High Performance Liquid Chromatography (HPLC), Agilent 1200 using an Aminex HPX-87H ion exclusion column at 45°C with 0.005 M H_2_SO_4 _eluent.

### Quantification of volatiles by PTR-MS

Proton transfer reaction-mass spectrometry (PTR-MS) was used to quantify a selected set of volatile compounds produced by *A. sarcoides *by constantly monitoring the gaseous effluent from the cultured reactor into the stationary phase of growth. The PTR-MS instrument ionized organic molecules in the gas phase through their reaction with H_3_O^+^, forming protonated molecules (MH^+^, where M is the neutral organic molecule) and fragment ions, which were detected by a standard quadrupole mass spectrometer. This process can be used with volatiles in air with or without dilution, since the primary constituents of air (nitrogen, oxygen, argon and carbon dioxide) have a proton affinity less than water and thus are not ionized. Most organic molecules (excepting alkanes) have a proton affinity greater than water and are therefore ionized and detected [Lindinger et al. 1998].

The cultured and un-inoculated controls were analyzed by diverting a portion of the reactor effluent gas to the PTR-MS. Measurements were made from the initial inoculation until 18.5 days. At times, the gas stream was diluted with additional air to prevent premature degradation of the MS detector and keep measurements within the linear dynamic range of the PTR-MS. During dilution periods, the dilution stream was added to the reactor effluent stream and balanced with the effluent flow rate using flow controllers. Sample lines were constructed from PFA Teflon tubing and stainless steel fittings. Mass spectral scans were acquired from 20 to 220 amu at 0.5 sec/amu.

The absolute concentration of constituents in a sample were quantified directly from the measured ion intensities using the known reaction time and the theoretical reaction rate constant for the proton transfer reaction [Lindinger et al. 1998]. Concentrations reported within were derived from the PTR-MS measurements and calculated using equations derived from reaction kinetics, assuming a reaction rate coefficient of 2 × 10^-9 ^ml s^-1 ^which is appropriate for the measured compounds [Ezra et al. 2004,Lindinger et al. 1998]. This method provided a means by which the measured ion intensity at any mass can be expressed as an equivalent estimate of concentration.

The total VOC concentration produced by the fungal culture is taken as the difference between the fungal culture and the control measurements. This total reflects only the contribution from those species having proton affinities greater than that of water, which is assumed to represent the majority of the VOCs produced.

### Volatile analysis with SPME GC-MS

Solid phase microextraction (SPME) was used to identify gaseous compounds as described previously [Griffin et al. 2010], but a brief description of the relevant details is provided here. Throughout the growth period, 12 mL of *A. sarcoides *culture was aseptically removed, sealed, centrifuged at 4,000 rpm for 4 minutes, then 10 mL of supernatant was added to a GC vial and stored at 4°C until analysis. For analysis, the SPME fiber (divinylbenzene/Carboxen on polydimethylsiloxane by Supelco) (Bellefonte, PA) was exposed to the vial headspace while the sample was stirred for 45 minutes. The fiber was inserted into the injection port of the GC at 240°C. The GC temperature was held at 40°C for 2 minutes, and then ramped to 230°C at 5°C min^-1^. The mass spectrometer was tuned to meet EPA Method 8260 BFB tuning criteria. The relative amount of identified compounds was estimated by comparison with a 4-bromofluorobenzene internal standard calibration curve delivered in methanol over the concentration range of 2 to 75 μg/mL.

The quantities of volatiles desorbed from the fiber were calculated from the peak area of the total ion current measured by the mass spectrometer. This method assumed that fungal VOCs and the internal standard have the same gas-liquid partitioning, efficiency of adsorption to the fiber, and response in the GC-MS system as that of the internal standard. As will be shown later, all of these conditions are not met, and the quantities obtained are estimates. Nevertheless, the use of the internal standard allowed for a relative quantitative comparison between samples from the same culture across a time period. Data acquisition and data processing were performed on Hewlett Packard ChemStation software system. The identification of compounds produced by *A. sarcoides *was made via library comparison using the National Institute of Standards and Technology (NIST) database, and all chemical compounds described in this report use the NIST Chemistry WebBook terminology [Linstrom and Mallard 2011]. When the library identified multiple compounds as matches, careful consideration of the spectra yielded one with much higher similarity or a determination of "unknown" was made.

### Henry's law constants

To characterize the headspace concentration response to a specific set of compounds at known initial concentrations using the PTR-MS and the SPME technique, a known mixture (Table 1) was introduced into the Biostat reactor system. The reactor was sparged with humidified air at 1.5 L/min, equivalent to that employed for the culturing conditions. Temperature (23°C) and stir rate (250 rpm) were held constant by the Biostat control system. A portion of the gaseous reactor effluent was diverted to the PTR-MS to measure effluent volatile concentrations over a time period of 48 hours to determine the Henry's Law constants for each volatile compound. Liquid aliquots were removed from the reactor at 0, 1, 2, 3, 6, 12, 24, and 48 hours for analysis by headspace SPME GC-MS.

**Table 1:** Initial concentrations and Henry's Law constants for compounds run as standards in the abiotic experiment

		Henry's Law Constants^a^		
**Standard Compound**	**C_o_^b ^(+/-)**	**NIST^c^**	**Yaws^d^**	**Experimental**

Acetaldehyde	2242 (415)	14	10.3	13.9

Benzaldehyde	2239 (23)	39	39.6	38.9

Ethanol	2822 (546)	120	121.7	173

Nonanal	2032 (851)	0.7	1.5	1.2

1-Octen-3-ol	1489 (653)	62^e^	39.4^e^	25.2

1-Butanol, 3-methyl	1294 (45)		73.8	77.6

^a ^All values in mol/kg*bar

^b ^Initial headspace concentration based on Henry's Law constants in ppbv with standard deviation in parenthesis

^c ^[Sander, R (2011)].

^d ^[Yaws, CL (1999)].

^e ^1-Octanol value, value for 1-Octen-3-ol not available

The relationship governing the equilibrium concentration in the liquid in relation to the headspace is defined by Henry's Law:

(1) $${\text{P}}_{\text{A}}=\frac{{\text{C}}_{{\text{A}}_{{\text{h}}_{\text{q}}}}}{{\text{H}}_{\text{A}}}$$

Where ρ_A _is the partial pressure of compound A, C_Aliq_, is its concentration in the liquid, and H_A _is the Henry's Law constant for A. Henry's Law constants were evaluated from the PTR-MS measurements following the methodology of [Karl et al. (2003)] and compared with literature values (Table 1) [Linstrom and Mallard 2011,Yaws 1999]. Experimentally determined Henry's Law constants were evaluated from the slope of the natural log of concentration versus time, and the initial substrate concentration was taken as the intercept. As the concentration of each compound decreases in the liquid, the headspace gaseous concentration in equilibrium with the liquid can be calculated from the first-order rate equation using the initial headspace concentration and the Henry's Law constant.

(2) $${\text{C}}_{\text{A}}(\text{t})={\text{C}}_{\text{Ao}}\exp\left[\frac{\cdot \text{F}}{{\text{H}}_{\text{A}}\text{VRT}}*\text{t}\right]$$

Where C_A _is the gaseous concentration of A, F is the flow rate of gas, V is the volume of liquid containing A, R is the ideal gas constant, and T is the temperature.

Initial concentration of compound A added to the reactor can be measured by PTR-MS and confirmed by calculations based on the volume of compound A added, V_A_.

(3) $${\text{C}}_{\text{Ao}}=\frac{{\text{V}}_{\text{A}}{\text{p}}_{\text{A}}}{\text{M}{\text{W}}_{\text{A}}{\text{H}}_{\text{A}}\text{PV}}$$

Where ρ_A _is the density of A, MW_A _is the molecular weight of A, and P is the pressure of the reactor headspace. The initial concentration for each standard from EQ. 3 is listed in Table 1.

## Results

### Abiotic results

**Figure 1:**
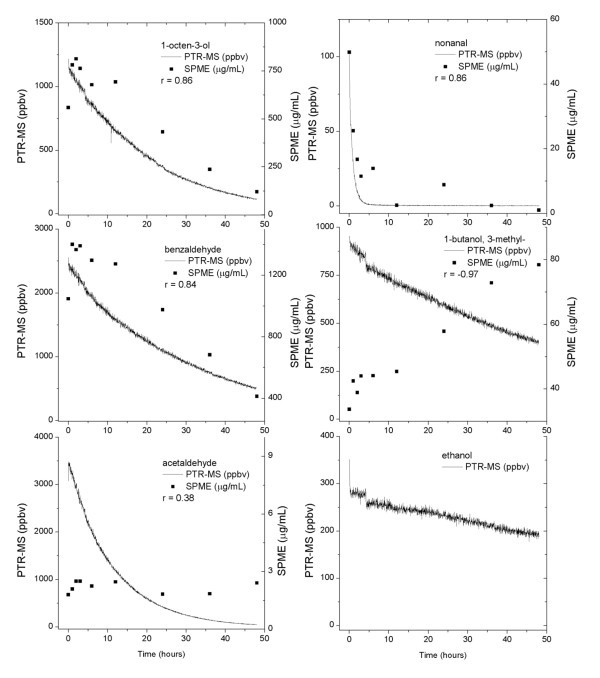
**Abiotic concentration profiles of standard compounds as measured by PTR-MS and SPME GC-MS**. Concentration profiles of each standard compound with abiotic measurements. PTR-MS response in parts per billion volume (ppbv) was monitored continuously over 48 hour time period. HS-SPME samples were removed at 0, 1, 2, 3, 6, 12, 24, 36 and 48 hours with correlation coefficients corresponding to six minute PTR-MS averages.

Abiotic results were obtained from known concentrations of six standard compounds in the reactor system under a set of controlled conditions that closely mimicked the fungal reactor system to better understand how the measurements of the PTR-MS and SPME GC/MS compare. All of the compounds were added at a level to achieve an initial headspace concentration of 2000 ppbv. The concentration profiles versus time for each of the standards as measured by the PTR-MS and the SPME fiber are shown in Figure 1. The headspace concentration of each measured standard decreased in an exponential fashion as predicted by Equation 2. Henry's Law, as an equilibrium relation, dictates that the concentration of each standard in the gas phase will decrease proportionally with time as the liquid phase concentration decreases due to sparging. The rate of decrease is determined by the air sparge rate and the system mass balance and is inversely related to the Henry's Law constant. For example, nonanal has the smallest Henry's Law constant reported in Table 1 and its headspace concentration decreased the fastest of all the analyzed compounds. Henry's law constants for each of the standards can be obtained by plotting the time series data from Figure 1 as the natural log of concentration versus time, yielding a linear relationship (not shown) where the slope of the line is related to the Henry's Law constant [Karl et al. 2003]. The Henry's Law constants calculated from the PTR data are consistent with those reported previously in the literature (Table 1), which indicates that the ions monitored are free from any interferences and the response of the PTR-MS is within the linear dynamic range of the instrument. The SPME results agree less favorably quantitatively and are discussed below. Qualitatively, the PTR-MS and SPME agree on the identification of the standard compounds with the exception of ethanol which was not detected by the SPME.

The SPME results in Figure 1 show that the divinylbenzene/Carboxen on polydimethylsiloxane fiber is inefficient at trapping acetaldehyde and ethanol. This result is not totally unexpected, as the fiber is not specified for the analysis of C2 compounds. It appears that the SPME fiber did not provide results proportional to the amount of acetaldehyde or ethanol present. Additionally, the SPME measurements of 1-butanol, 3-methyl- had a contradictory trend to the PTR-MS measurements and did not decrease in concentration over time. The SPME results for the higher molecular weight components appear to more closely follow the anticipated Henry's law behavior and are fairly consistent with the PTR-MS results with correlation coefficients above 0.8.

**Figure 2:**
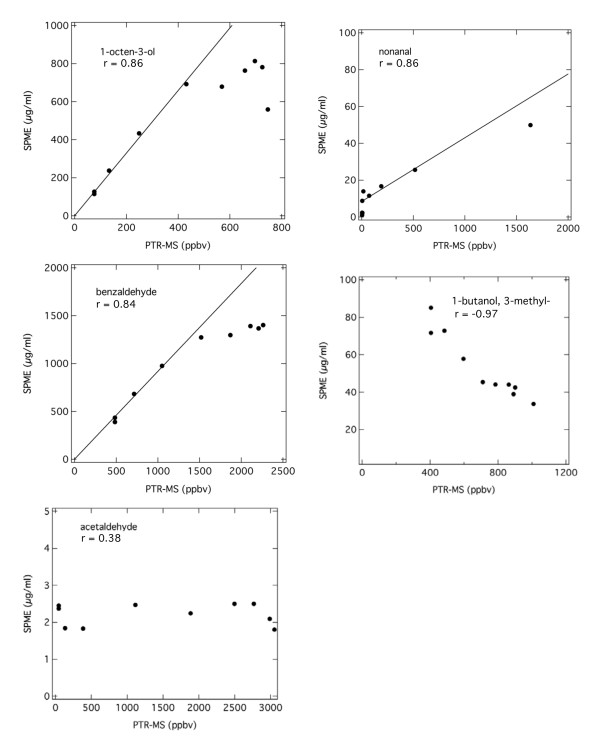
**Abiotic correlation of standard compound concentrations**. Correlation of relative concentration measurements for PTR-MS and the SPME GC-MS technique for all compounds with abiotic measurements except for ethanol, which had a non-detect response by SPME GC-MS. PTR-MS values are centered at SPME sample collection times and are six minute averages. Lines indicate linear correlation at low concentrations with divergence at high concentrations while correlation coefficients include all data points.

A better means of accessing the correlation between the PTR-MS and SPME data can be made by collecting the concentration data at the same discrete time points. The PTR-MS and SPME time point concentrations have been plotted against each other in Figure 2 with the corresponding correlation factors. Note that ethanol was not included in Figure 2, as there was no ethanol peak detected in the SPME GC-MS technique. While it is apparent in both Figures 1 and 2 that the measurement of acetaldehyde and 1-butanol, 3-methyl- by the two techniques do not correlate well, Figure 2 shows that the degree of correlation for benzaldehyde and 1-octen-3-ol is concentration dependent. The PTR-MS and SPME measurements appear to track one another at the lower concentrations. At the higher concentrations, the SPME results appear to saturate, which suggests that the adsorption capacity of the fiber may have been exceeded. The nonanal results appear to be similarly affected, but the comparison is less definitive due to rapid removal of nonanal from solution and the appearance of the non-zero intercept. The contradictory trend for 1-butanol, 3-methyl- suggests that the adsorption process is competitive, because the 1-butanol, 3-methyl-concentration, as measured by SPME, increases as the stronger adsorbing species decrease in concentration as they are removed from solution. These results indicate that the concentrations measured by the SPME technique are strongly dependent not only on the type of mixture constituents, but also on the relative concentrations of those components. Thus even in relatively simple systems such as this, some care must be exercised in deducing quantitative information from the SPME data.

### Quantification of volatile products of *A. sarcoides*

**Figure 3:**
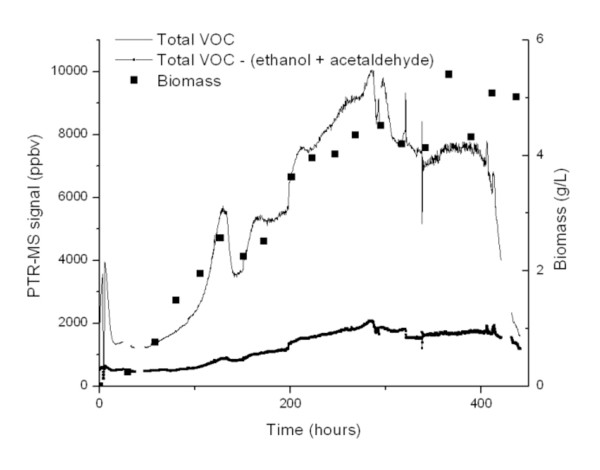
**Total VOCs in sparged air from *A. sarcoides *culture as measured by PTR-MS**.The total VOCs excluding the signals from ethanol and acetaldehyde were near 2000 ppbv at their maximum. Biomass was measured by dry weight.

Applying these techniques to the biological system, Figure 3 shows the time series of the total VOC signal (sum of all the ions from m/z 40-205) as measured by the PTR-MS along with a measure of *A. sarcoides *biological growth expressed as biomass density. The initial variation in the VOC signal is due to inoculation of the reactor with an active culture, and the initial peak decays away due to the sparging process. The culture demonstrated a lag phase until day 3 before entering an exponential growth phase. The total VOC concentration increased as the fungus entered exponential phase indicating growth associated product synthesis. The flow rate of gas supplied to the reactor was constant such that increasing concentrations demonstrates increased metabolic production of VOCs. The maximum ion concentration of VOCs measured was 10,000 ppbv at 284 hours (near 12 days). The cell concentration began to stabilize on day 12 around 4.5 g/L indicating the culture's entry into stationary phase. Once the stationary phase was reached after day 12, the total VOC signal stabilized, and then dropped rapidly around 380 hours. Throughout stationary phase, the PTR-MS measured the VOCs excluding acetaldehyde and ethanol at 2,000 ppbv. The very sharp transitions in the VOC signal are due to variations in the sparge flow and should not be interpreted as specific metabolic changes.

**Figure 4:**
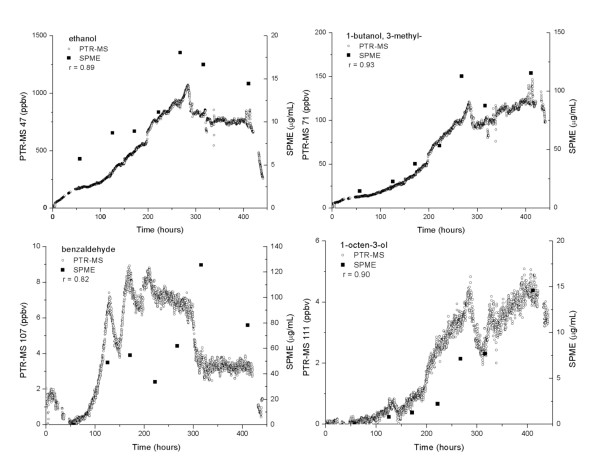
**Measured concentrations of ethanol, 1-butanol, 3-methyl-, benzaldehyde and 1-octen-3-ol over the course of the growth period**. Response of ethanol, 1-butanol, 3-methyl-, benzaldehyde and 1-octen-3-ol over the course of the growth period as measured by PTR-MS and HS-SPME GC-MS including correlation coefficient, r.

Continuous monitoring by the PTR-MS revealed that two ions (m/z 45 (acetaldehyde) and m/z 47 (ethanol)) comprised the majority of the ion signal from the *A. sarcoides *culture. Excluding m/z 45 and m/z 47, the remaining ion intensity measured was between 1,000 and 2,000 ppbv in concentration for the majority of the time (Figure 3). The remaining ions account for ~ 14% of the total VOC signal. A listing of all of the ions whose intensity exceeded 1 ppbv is tabulated in Table 2 along with their signal intensity at discrete time points. Ions of the carbon 13 isotopes are not included, i.e. m/z 72 which is 5.5% of m/z 71. Total production of compounds in Table 2 increased to over 2,000 ppbv at 280 hours and leveled off as the fungus entered stationary phase after day 12. The majority of these compounds were composed of 4 and 5 carbon alcohols, acetic acid or acetate esters and compounds producing m/z 59 (acetone and/or propanal). The PTR-MS signal from 1-butanol, 3-methyl- was over 1% of the total VOCs. Notable compounds of fuel interest included 1-octen-3-ol, nonanal, 1-butanol, 3-methyl- and benzaldehyde (Figure 4). The PTR-MS signals of the alcohols in Figure 4 increased steadily until near the end of the growth cycle with a peak around 275 hours at the end of the exponential growth phase. The benzaldehyde signal reached a maximum around 210 hours before halving just before 300 hours and remained there for the stationary growth phase of *A. sarcoides*. The SPME measured concentration profiles correspond well with the PTR-MS results for the higher molecular weight alcohols (correlation coefficients > 0.8), but not for ethanol or benzaldehyde (correlation coefficients < 0.7). An increase in the concentration of a compound should elicit a relative increase in the measured response of each technique. Figure 4 and Table 2 clearly show that there are compounds for which the SPME response to a change in VOC concentration does not scale with the corresponding PTR-MS signal change. The differences in response show the confounding effects of SPME fiber selectivity.

**Table 2:** Select ion signals in the dynamic headspace of *A.sarcoides *culture measured by PTR-MS

Mass (amu)^a,b^	Signal Intensity (ppbv) at given age in days^c^						
	
	2	5	9	11	13	15	17
33 [methanol]	1.0	3.6	6.8	9.1	11.1	10.6	12.8

41	6.3	12.7	27.1	40.8	48.8	49.7	52.2

43	36.6	87.8	198	264	251	260	269

57	11.1	44.7	129	180	187	201	234

59 [C_3_H_6_O]	17.0	71.9	198	264	251	260	269

61	7.6	23.2	69.6	87.7	60.4	54.8	52.5

69	1.7	3.5	5.9	7.5	6.6	7.3	8.6

71 [1-butanol, 3-methyl-]	12.9	24.0	76.4	99.1	98.6	111	127

73 [C_4_H_8_O]	4.4	39.8	38.6	41.3	43.8	44.1	38.2

75	0.2	0.5	1.9	2.3	1.4	1.5	1.6

87 [C_5_H_10_O]	1.0	2.2	4.0	4.9	7.4	10.0	8.0

89 [ethyl acetate]	5.3	14.1	47.9	53.4	32.0	31.0	31.6

91	0.3	0.8	2.0	2.6	2.0	2.1	2.0

93	0.2	0.2	0.9	1.1	0.6	0.8	0.9

105	0.2	0.5	1.8	2.4	2.1	2.2	2.6

107 [benzaldehyde]	0.2	6.3	7.5	6.9	3.5	3.4	3.3

111 [1-octen-3-ol]	0.1	0.5	2.5	3.4	2.6	3.8	4.4

115	0.0	0.5	1.8	1.8	0.9	0.5	0.7

117	0.2	0.4	1.5	2.1	2.0	3.0	4.2

129	0.1	0.4	0.9	1.3	1.2	1.2	1.5

143 [nonanal]	0.1	0.6	0.6	0.7	0.2	0.4	0.5

SUM	106	338	847	1086	1013	1063	1153

^a ^Acetaldehyde (45), ethanol (47) and minor ion signals are excluded.

^b ^Tentative assignments to the ion are given in brackets.

^c ^Signal intensity denoted as 4 hour averages associated with the data in Table 3.

The total ion signal was consistent with *A. sarcoides *biomass concentration; meaning VOC production was closely related with growth (Figure 3). The VOC concentration profiles vary by compound as shown in Figure 4 with some being associated with growth. The compounds with the highest concentrations were acetaldehyde and ethanol. The major ion signatures, m/z 101, m/z 119, and m/z 159 found in the [Strobel et al. (2008)] experiment on oatmeal agar were not observed in this study. This change in product composition is most likely due to different carbon source and experimental conditions.

### Identification of volatile products of *A. sarcoides*

The qualitative information provided by the SPME technique identifies specific VOCs, thereby allowing better ion assignments to be made with the PTR-MS. Most of the compounds positively identified by the SPME technique were also detected with PTR-MS, though the relative amounts of the identified compounds differed significantly between the two techniques. In some instances, the ions detected by PTR-MS were not unique and reflect the collective signal from more than one compound; often the case for lower mass ions. A total of twenty-seven distinct peaks were found using SPME GC-MS, with eight being unidentifiable (Table 3). Of the identified compounds, there was a mixture of (in order of descending frequency) organic acids and esters, alcohols, aldehydes, aromatics and alkanes. Two alkanes were identified by the SPME GC-MS (pentane and 4-methyl-heptane). The branched 8 carbon alkane, 4-methyl-heptane, was produced from Day 2 to Day 15. Physical property information for Table 3 compounds is listed in Additional file 1.

**Table 3:** GC-MS head-space analysis of VOCs produced by *A.sarcoides *culture collected with a SPME fiber

Retention	Possible Compound^a^		Peak Area at given age in days^b^			
Time (min)	(Molecular mass (Da))	2	5	9	15	17
1.34	Unknown (56)	17.8	13.0	ND	ND	ND

1.38	Pentane (72)^c^	ND	12.6	38.7	22.6	31.8

2.01	Heptane, 4-methyl- (114)^d^	12.9	12.6	11.1	15.6	ND

2.09	Unknown (118)	10.2	9.1	7.3	8.8	6.7

3.26	Ethyl acetate (88)^c^	10.1	19.5	52.9	51.8	44.8

3.89	Ethanol (46)^d^	79.3	121.8	157.3	223.5	188.2

6.07	Unknown (114)	ND	16.1	27.5	45.2	ND

6.40	1-Propanol, 2-methyl- (74)^c,d^	10.1	20.1	17.7	74.9	52.8

7.70	Unknown	11.7	11.0	7.7	11.0	23.7

8.19	1-Butanol, 3-methyl- (impure) (88)^c,d^	88.6	139.7	334.1	665.5	667.6

9.15	Unknown (134)	8.0	10.7	12.8	ND	ND

9.56	Unknown (159 or 88)	ND	11.7	18.7	35.8	ND

11.65	1-Octen-3-ol (128)^c,d(isomer)^	ND	7.6	22.8	80.3	84.5

12.02	Acetic acid, octyl ester (172)^c^	15.6	7.8	3.4	2.6	4.4

12.95	Benzaldehyde (106)	5.2	302.8	211.3	679.7	453.5

13.33	Acetic acid, nonyl ester (186)^c^	33.9	32.8	7.5	5.5	ND

13.99	Unknown (122)	10.9	32.8	1.7	ND	ND

14.58	Acetic acid, n-decyl ester (200)^c,d^	19.3	9.1	ND	ND	ND

14.86	C_15_H_24_, sesquiterpene (204)^c^	19.3	8.1	5.4	4.1	ND

15.95	Nonanal (142)	15.1	12.1	12.3	17.6	46.7

16.99	Benzyl alcohol (108)^c,d^	ND	5.1	39.3	39.1	34.9

18.37	Unknown (126)	4.4	5.1	10.0	17.0	26.8

18.71	Decanal (156)	ND	ND	ND	24.7	60.8

23.27	Butanoic acid, 3-methyl- (102)^c^	ND	ND	ND	8.9	20.3

24.83	Oxime-, methoxy-phenyl- (151)	ND	ND	ND	39.8	35.7

26.30	Acetic acid, 2-phenylethyl ester (164)^c^	ND	ND	ND	24.5	24.0

28.33	Phenylethyl alcohol (122)^c,d^	ND	ND	ND	37.6	36.6

^a ^Unknown compounds represent those whose assignment is uncertain. However, probable molecular masses are included when consistent.

^b ^Peak areas listed as 10^6^; ND = signal not detected

^c ^identified by Griffin et al. 2010 (12)

^d ^identified by Strobel et al. 2008 (32, 33)

As noted in Table 3, twelve compounds identified in this study were also detected by [Griffin et al. (2010)] on various media including cellulose. Although there were major differences in the volatile constituents identified, this was not surprising due to the very different experimental conditions of this study. For example, acetic acid, heptyl ester was reported [Strobel et al. 2008] as one of most abundant compounds, but it was not identified by SPME in these results. However, many minor compounds identified by [Strobel et al. (2008)] on oatmeal agar were identified in this study despite conservative identification changes later made [Strobel et al. 2010]. These compounds are noted in Table 3 and include heptane, 2-methyl-; acetic acid, decyl ester; ethanol; 1-propanol, 2-methyl-; phenylethyl alcohol; 1-butanol, 3-methyl-; and an isomer of 3-octen-2-ol (1-octen-3-ol).

Of the compounds identified by SPME GC-MS, the concentration profiles of several correlated with the PTR-MS measured concentrations with correlation coefficient exceeding 0.80 (Tables 2 and 3). These compounds include acetic acid, ethyl ester (89) and the alcohols: 1-butanol, 3-methyl- (71), 1-octen-3-ol (111) (Figure 4). The concentration profile of 1-butanol, 3-methyl- as indicated by SPME correlated well with the PTR-MS measurements of ion 71 (Figure 4) with a correlation coefficient of 0.93, despite the negative correlation found by the abiotic results (Figures 1 and 2). A possible explanation for this is the concentrations of other compounds in the fungal gas phase were small and did not preferentially displace 1-butanol, 3-methyl- from the fiber, while in the abiotic results, this was not the case. The concentration profiles for 1-octen-3-ol were strikingly similar despite the relatively low concentration of this product. This was also the case for butanoic acid, 3-methyl- and nonanal whose PTR-MS measured concentrations were less than 1 ppbv. Like 1-butanol, 3-methyl-, their measured concentrations steadily increased through the majority of the time points. The variance in reported signals is seen in Tables 2 and 3 and Figure 4.

The SPME measurements of compounds that did not correlate well with the PTR-MS signal included benzaldehyde (107) and 1-propanol, 2-methyl- (57). For benzaldehyde, the first SPME data point matches the PTR-MS signal, but afterwards, the measured SPME concentrations are out of step with PTR-MS concentrations (Figure 4). There was a similar incongruity with the 1-propanol, 2-methyl- measurements. The PTR-MS measurement of 1-propanol, 2-methyl- is made by monitoring the m/z 57 ion. The m/z 57 ion, however, is not unique to 1-propanol, 2-methyl-, and it is probable that the lack of agreement between the PTR-MS and SPME results may be due to signal interference in the PTR-MS measurement. While perturbations in the PTR-MS signal for benzaldehyde could be similarly explained by signal interference, but the abiotic results for benzaldehyde showed no interference, eliminating this possibility.

## Discussion

Taken together, the results of the PTR-MS and SPME GC-MS techniques provide a more definitive description of the VOCs produced by *A. sarcoides *for product (e.g. biofuel) development. However, the challenges associated with the quantitative and qualitative analysis of complex VOC mixtures in an aqueous solution are non-trivial, and neither method had the ability to both identify and quantify the breadth of compound diversity synthesized by this complex fungal system.

The *A. sarcoides *culture consistently produced numerous VOCs. There was little quantitative agreement in VOC concentrations between the SPME technique and the PTR-MS for the suite of compounds produced by *A. sarcoides*. Both techniques have advantages for exploring potential fuel products from fungal or other cultures. SPME effectively identifies a range of compounds produced by the culture, and at modest concentrations, has adequate fiber capacity for many relevant compounds. The use of PTR-MS gives quantitative data on the evolution of volatiles permitting temporal resolution of microbial volatile production. These data can be used to help elucidate real-time metabolism shifts of an organism for product applications (excluding alkanes) and metabolic modeling. The PTR-MS results may also be valuable in guiding genetic engineering efforts to improve production rates of specific compounds..

To effectively use PTR-MS and SPME GC-MS, the inherent differences in signal acquistion must be considered. Foremost, PTR-MS is a continuous measurement of VOCs in the gaseous stream stripped from an aqueous culture; SPME is a measurement of the headspace compounds that develop above a liquid aliquot after a 45 minute exposure and equilibration period. However, SPME is not truly an equilibrium measurement, because the concentration of the headspace is potentially being depleted by compounds adsorbing to the fiber, lowering their vapor pressure and thereby constantly shifting the headspace composition (Eq. 1) [Zeng and Noblet 2002]. SPME adsorbs VOCs from the headspace then estimates a liquid concentration based on one internal standard. Therefore, we anticipate differences in distribution of VOCs detected by the two methods (Figures 1 and 2). The measurement variability that exists between the two techniques should decrease if the gaseous effluent measured by the PTR-MS is at or near equilibrium with the compounds in the liquid. Based on the abiotic experimental results with a known mixture of VOC standards, equilibrium was assumed for the experimental reactor system.

A limitation of PTR-MS is that alkanes are not ionized by H_3_O^+ ^and therefore are not detectable making it unsuitable for some biofuel applications. Additional quantitative complications can arise due to the presence of fragment ions (decomposition products of the protonated molecule). While ionization via proton transfer with H_3_O^+ ^is considered to be a relatively soft ionization method, many of the components produced by *A. sarcoides *fragment extensively. Most notable are the alcohols that, except for methanol, fragment by the elimination of H_2_O from the protonated molecule [Buhr et al. 2002]. Thus, the alcohols are detected as an ion corresponding to (M-OH)^+^. Despite these considerations, the results clearly demonstrate the applicability of the PTR-MS for time series resolution of volatiles from biological cultures.

SPME analysis should reflect the chemical composition and capacity on the SPME fiber if possible. However, not all compounds adsorb to the fiber material with equal affinities or at equal rates, such that the use of one internal standard is not sufficient to determine all constituent concentrations [Pawliszyn 1997]. The preferential adsorption by some compounds appears to be further exacerbated if the capacity of the fiber is exceeded. The least volatile, highest affinity compounds will be extracted from the headspace-liquid matrix last, therefore adequate extraction times must be observed [Pawliszyn 1999]. Therefore, drawing conclusions about the concentration of constituents in complex mixtures is very difficult due to fiber selectivity and saturation characteristics. In general, the SPME fiber appeared to preferentially adsorb the higher molecular weight compounds and therefore over predicts their abundance (Table 3). While these ions may have been detected by the PTR-MS, signals were low in concentration either because their headspace concentrations were low or because of possible adsorptive losses in the sample lines and therefore were not included in Table 2. An outcome of this preferential adsorption was seen in the 1-butanol, 3-methyl- data. In the fungal culture, the SPME data correlated well with the PTR-MS signal (Figure 4), but not in the abiotic data (Figure 1) because the culture headspace environment was dominated by lower molecular weight compounds. The negative trend seen in the abiotic results was most likely because the higher molecular weight compounds were preferentially adsorbed by the SPME fiber, leaving few adsorption sites for the other compounds (Figure 1). The extent of fiber bias for the lower volatility, higher molecular weight compounds is not easily predicted or measured for complex mixtures. However, SPME appears to be more applicable in the fungal headspace environment than in conditions where higher concentrations of higher molecular weight compounds were present, such as in the abiotic system.

The PTR-MS and SPME results demonstrated the quantitative limits of the SPME technique even when an internal standard was used. However, use of SPME to estimate relative concentrations of higher molecular weight compounds may be reasonable if application is restricted to qualitative temporal trends and not individual quantitative time points. A possibility for improving the SPME technique is to use replicate samples with additional fiber types to better capture and characterize the breadth of compounds present.

This study shows that despite their shortcomings, the combined use of PTR-MS with a qualitative technique such as GC-MS by headspace SPME is a valuable method for obtaining more detailed analysis of the VOCs produced by microbial systems and can be applied to biofuel product development. However, when quantifying compounds as produced by metabolic processes, especially when those compounds are in a complex mixture, the combination of multiple techniques is warranted until a single more robust qualitative and quantitative analytical method is developed.

## Abbreviations

GC-MS: Gas Chromatography - Mass Spectrometry; NIST: National Institute of Standards and Technology; PTR-MS: Proton Transfer Reaction - Mass Spectrometry; SPME: Solid Phase Microextraction; VOCs: Volatile Organic Compounds.

## Competing interests

The authors declare that they have no competing interests.

## Supplementary Material

Additional file 1 **Physical properties and ions of Table **3**compounds**. Table 3 compounds are listed with their PTR-MS ions including fractions, Henry's Law constant, boiling point and vapor pressure. Click here for file
