# Supplementary material for: Resolution of volatile fuel compound profiles from Ascocoryne sarcoides: a comparison by proton transfer reaction-mass spectrometry and solid phase microextraction gas chromatography-mass spectrometry
Source: AMB Express. 2012 Apr 5;2:23. doi: 10.1186/2191-0855-2-23 (PMC3402149; doi:10.1186/2191-0855-2-23)
Supplement: Additional file 1 — Physical properties and ions of Table 3compounds. Table 3 compounds are listed with their PTR-MS ions including fractions, Henry's Law constant, boiling point and vapor pressure. [file 2191-0855-2-23-S1.PDF]

Additional file 1. Physical properties and ions of Table 3 compounds.

**Compound**	**[+]Boiling Point (^{o}C)**	**[+]Vapor pressure (mmHg at 25°C)**	**[*]Henry's Law Constant (mol/kg*bar)**	**PTR-MS ions (fractions)**
pentane	36	527	$7.8x10^{-4}$	No Reaction
4-methy-heptane	117	20.5	$2.7x10^{-4}$	No Reaction
acetic acid, ethyl ester	77	112	8.9	[a]43(34), 61(43), 89(24)
ethanol	79	[#]58.7	120	[a]29, 47
2-methyl-1-propanol	108	[#]10.3	100	[a]57(100)
1-butanol, 3-methyl	131	4.76	81	[a]41(11), 43(35), 71(37)
1-octen-3-ol	[+]174	.513	39	[b]41(4), 57(3), 69(45), 111(42), 129(6)
acetic acid, octyl ester	210	.194		[b1]43, 61
benzaldehyde	179	1.27	[e] 39	[c]107(100)
acetic acid, nonyl ester	227	.197		[b1]43, 61
acetic acid, decyl ester	244	.031		[b1]43,61
alpha amorphene	[+]271	.0107		[a1]81, 95, 109, 123, 135, 149, 205
nonanal	191	.532	0.69	[d]69(21), 83(10), 125(18), 143(51)
benzene methanol	205	.094	9000	[d]79(15), 91(85)
decanal	209	.207		[d1]157
3-methyl-butanoic acid	175	.554	1200	Not measured
Methoxy-phenyl-oxime	254	~.023		Not measured
acetic acid, 2-phenylethyl ester	239	.0564		[a]43(37), 61(63)
phenylethyl alcohol	219	.074	[#]349.5	[a]105(100)

[*]Sander 2011
[+]http://www.thegoodscentscompany.com/data/rw1024051.html
[#] Yaws 1999
[a] Ezra et al. 2004
[a1] ions observed for the sesquiterpene carophyllene
[b] Buhr et al. 2002
[b1] predicted ions based data reported for smaller acetate esters
[c] Warneke et al. 1996
[d] This study
[d1] insufficient ion intensity to identify fragment ions.
[e] 1-Octanol value
